# Ellagic acid protected the gingival tissue via fibroblast and epidermal growth factors in rats

**DOI:** 10.1590/acb391224

**Published:** 2024-03-11

**Authors:** Gülüçağ Giray Tekin, Buşra Deveci, Engin Deveci

**Affiliations:** 1 Batman University – Faculty of Dentistry – Department of Periodontology – Batman, Turkey.; 2 Diyarbakir Oral and Dental Health Center – Diyarbakir, Turkey.; 3 Dicle University – Medical School – Department of Histology and Embryology – Diyarbakir, Turkey.

**Keywords:** Ellagic Acid, Gingiva, Epidermal Growth Factor, Fibroblast Growth Factors, Antioxidants, Rats

## Abstract

**Purpose:**

To investigate the effect of ellagic acid (EA) in gingival tissues injury in rats.

**Methods:**

Twenty rats were categorized into two groups. In burn group, an excisional wound area was created by removing a 4-mm diameter flap from the left molar region in the mucoperiosteal region of the gingiva. In burn + ellagic acid group, 1.2 mg/mL EA was administered as irrigation for one week. Animals was sacrificed under anesthesia at the end of experiment. Malondialdehyde (MDA), myeloperoxidase (MPO) and glutathione (GSH) level were measured. Hematoxylin and eosin, fibroblast growth factor (FGF) and epidermal growth factor (EGF) immunostainings were applied to tissues.

**Results:**

MDA, MPO, inflammation and leukocyte infiltration were high in burn group. Degeneration epithelium, edema and inflammatory cell infiltration in connective tissue areas, and dilatation and congestion in blood vessels were observed in burn group. In burn + EA group, the gingival epithelium improved, collagen fiber production increased and organized dermis were observed. After burn injury, FGF and EGF activity was increased in EA treated groups.

**Conclusions:**

We suggest that EA have the potential for better healing outcomes in oral wounds. EA seems to have promising therapeutic efficacy to enhance oral wound healing.

## Introduction

Current therapies for oral wound healing lack effective treatment outcomes in oral wound management and tissue regeneration. The lack of successful therapy for oral mucosal wounds has forced clinicians to find out alternative treatments and potential therapies to facilitate intraoral healing.

Following injury, the oral mucosa undergoes several biological healing processes for homeostasis. While wound healing is known and treated in cutaneous wounds, there is limited literature in intraoral healing, complicating clinical treatment alternatives. Regenerative approaches have committed for the enhance oral wound healing, but they need different effective options to promote tissue re-epithelialization and extracellular matrix (ECM) remodeling^1^. Remodeling of the granulation tissue occurs and that provides pro-regenerative growth factors like fibroblast growth factor (FGF), epidermal growth factor (EGF), and vascular endothelial growth factor^2^. Fibroblasts migrate to the provisional matrix and are integral for ECM remodeling; these cells lay down matrix proteins, including collagen and fibronectin, to provide structural integrity of the healing tissue^3^. When the body is unsuccessful to build homeostasis post-injury, the phases of wound healing are disrupted and result in impaired tissue regeneration. This is when the inflammation extends greater than seven days. So,delayed epithelialization and tissue necrosis becomes^3^. Impaired wound healing can occur from continued secretion of pro-inflammatory mediators and can be characterized by granuloma formation, fistula occurrence, wound dehiscence, ulcers, and excessive bleeding^4^.

Ellagic acid (2,3,7,8-tetrahydroxy-chromeno[5,4,3-cde]chromene-5,10-dione) (EA) is a bioactive polyphenolic compound (tannin), with a molecular weight of 302 gmol^-1^. EA shows a wide range of biological activities and potential beneficial effects on human health^5^. The presence of EA in various commercial products giving antioxidant activity has also been reported. EA has a variety of benefits for its anti-mutagenic, antimicrobial and antioxidant properties^6^^–^ 8. It prevents the formation of tumors, interact with the cell’s walls or sites easily to complex proteins, preventing the proliferation of metastatic cells. Losso et al.^9^ revealed the potential cytotoxic and anti-proliferative activities of EA in human cells, lung, colon, breast, and prostate cancer, and suggested that doses of 1 to 100 μmol/L inhibited the proliferation of the cancer cells. EA possibly causes apoptosis in cancer cells by inhibiting factors that promote metastasis. EA is a chemo preventive agent in cancers caused by polycyclic aromatic hydrocarbons and was approved to prevent a continuous tumor growth in cancer cells with the induction of apoptosis or cell death^10^. EA shows its antioxidant properties, by eliminating free radicals.

EA as antiviral and antimicrobial agent can inhibit the growth of pathogens in humans, probably coupled with protein in bacteria walls, such as that of *Bacillus*, *Staphylococcus*, and *Salmonella* 6. EA prevents eye, kidney, heart and joints of upper and lower extremities damage, caused by high blood glucose levels^11^ with its ability to inhibit the enzyme aldolase reductase, responsible for the production of proteoglycan type compounds in small blood vessels, causing blindness, kidney damage, paralysis, heart attacks and loss of limbs associated with the two types of diabetes. EA seems to increase the insulin activity and has several inflammatory effects and oxidative stress reduction^12^^,^ 13.

In this experimental model, we aimed to investigate the potential of EA for tissue regeneration in wounds of gingival tissues.

## Methods

### Animals

All experimental protocol was approved by the Local Ethical Committee of Animal Experiments of Dicle University, Turkey. The number of rats was calculated with the Gpower3.1 program, effect size = 0.8, power = 0.60, alpha = 0.15. Forty-eight 12-week-old Sprague Dawley rats were fed in stainless steel cages at 22 ± 2°C with normal diet and tap water for 12 hours in light and 12 hours in darkness without any restriction.

### Surgery protocol

Using 90-mg/kg ketamine hydrochloride and 8-mg/kg xylazine (intramuscular) under general anesthesia, sterilization with Povidone iodine solution was provided, and an excisional wound area was created by removing a 4-mm diameter flap from the gingiva located in the left molar region. Irrigation agents were started to be applied in the form of 1 cc irrigation solution after trauma was created and were given as 30-second applications once a day at the same time every day. Buprenorphine was used as a post-traumatic analgesic. A single dose of 0.01-mg/kg subcutaneous was administered for postoperative analgesia. EA (catalog no: E2250) was imported from Merck (Germany). Irrigation solutions was prepared as 1.2 mg/mL in 1 cc.

### Experimental design

- Burn group: The animals in this group were injured in the palate and gingiva, and sacrificed by intracardiac blood collection under anesthesia;
- Burn + EA group: Burn was created on the palate and gingiva of the animals in this group, and 1.2-mg/mL EA was administered as irrigation for one week. Animals were sacrificed by intracardiac blood draw under anesthesia.

The following pilot study protocols were used to administer EA as an oral irrigation solution^14^^,^ 15.

### Biochemical analyses

EA was applied locally as an irrigator solution. Since it is absorbed in the digestive tract, we looked at the levels of malondialdehyde (MDA), myeloperoxidase (MPO), and glutathione (GSH) in the blood. Blood samples were collected in tubes with a gel separator and centrifuged for 5 minutes at 1,550 g. The supernatant plasma was removed and placed in polypropylene plastic tubes. The tubes were properly labeled with the appropriate sample name and type. Samples were taken and stored at -80°C for the determination of MDA, GSH and MPO. MDA levels were determined using the double heating method by Draper and Hadley^16^. MDA values were expressed as nanomoles per gram (nmol/g) of wet tissue. The GSH activity was measured by Paglia and Valentine’s^17^method. Data were expressed as U/g protein. MPO activity in tissues was measured by a procedure similar to that described by Hillegass et al.^18^. MPO is expressed as U/g tissue.

### Histopathological analysis

Gingival sections were obtained for histopathological analysis and fixed in 10% buffered formalin, dehydrated in ethanol (50 to 100%), purified in xylene, and embedded in paraffin. Sections (4–5-mm thick) were cut and stained with hematoxylin and eosin (H&E), and studied to assess the pathological changes in the gingiva tissue^19^.

### Immunohistochemical analysis

Formaldehyde-fixed tissue was embedded in paraffin wax for further immunohistochemical examination. Sections were deparaffinized in xylene and passed through descending alcohol series. The antigen retrieval process was performed in citrate buffer solution (pH 6) for 15 minutes in a microwave oven at 700 W. Sections were allowed to cool at room temperature for 30 minutes and washed twice in phosphate buffered saline (PBS) for 5 minutes. Endogenous peroxidase blockage was performed in a 3% hydrogen peroxide solution for 7 minutes. The washed samples were incubated in Ultra V block (catalog no. TA-015UB, ThermoFischer, United States of America) for 8 minutes. Blocking solution was removed from the sections and allowed to incubate overnight at +4°C with primary antibodies EGF (catalog no: ab9695, Abcam, United States of America), and FGF (catalog no: ab92337, Abcam, United States of America). After washing the sections in PBS, secondary antibody (TP-015-BN, ThermoFischer, United States of America) was applied for 20 min. The sections were washed in PBS for 2 × 5 min and then exposed to streptavidin-peroxidase (TS-015-HR, ThermoFischer, United States of America) for 20 min. Sections washed with PBS were allowed to react with 3,3′-diaminobenzidine (DAB) (TA-001-HCX, ThermoFischer, United States of America) chromogen. Counterstaining with hematoxylin was applied, and, after washing, the preparations were mounted. Sections were examined under a light microscope (Zeiss Imager A2, Germany)^20^^,^ 21.

### Statistical analysis

Statistical analysis was performed by the IBM Statistical Package for the Social Sciences 25.0 software (IBM, Armonk, New York, United States of America). Data distribution was analyzed through Shapiro-Wilk’s test. The data were recorded as median (minimum–maximum) with mean rank value. Binary group comparisons were evaluated with Mann-Whitney’s test. P < 0.05 was accepted as the significance level.

## Results

### Biochemical findings

Statistical analysis of biochemical (MDA, MPO and GSH) and histological parameters (epithelization, inflammation, leukocyte infiltration, FGF and EGF expression) were shown in Table 1. Compared to control group, GSH, epithelization, FGF and EGF expression were statistically increased. MDA, MPO, inflammation, and leukocyte infiltration were significantly lower in burn + EA group than in burn group (Table 1).

**Table 1:** Biochemical and histological parameters of burn and burn + ellagic acid groups. Data distribution was analyzed through Shapiro-Wilk’s test. The data were recorded as median (minimum–maximum) with mean rank value. Binary group comparisons were evaluated with Mann-Whitney’s test. P < 0.05 was accepted as the significance level.

Parameters	Groups	n	Median (min–max)	Mean rank	P-value
MDA	Burn	10	47.45 (33.32–68.27)	14.50	P = 0.003
	Burn + ellagic acid	10	35.0 (20.74–43.09)	6.50	
MPO	Burn	10	1.75 (102–1.83)	14.50	P = 0.012
	Burn + ellagic acid	10	0.73 (0.24–1.76)	6.50	
GSH	Burn	10	4.48 (3.07–6.39)	6.50	P = 0.004
	Burn + ellagic acid	10	7.32 (3.91–9.72)	14.40	
Epithelization	Burn	10	2.00 (1.00–4.00)	7.40	P = 0.009
	Burn + ellagic acid	10	4.00 (2.00–4.00)	13.50	
Inflammation	Burn	10	4.00 (0.00–4.00)	12.25	P = 0.004
	Burn + ellagic acid	10	2.00 (1.00–4.00)	8.75	
Leukocyte infiltration	Burn	10	4.00 (2.00–4.00)	12.10	P = 0.003
	Burn + ellagic acid	10	2.00 (1.00–4.00)	9.20	
FGF expression	Burn	10	2.00 (1.00–4.00)	7.70	P = 0.013
	Burn + ellagic acid	10	3.50 (2.00–4.00)	13.20	
EGF expression	Burn	10	3.00 (1.00–3.00)	7.50	P = 0.022
	Burn + ellagic acid	10	4.00 (2.00–4.00)	13.40	

MDA: malondialdehyde; MPO: myeloperoxidase; GSH: glutathione. Source: Elaborated by the authors.

### Histopathological findings

#### Hematoxylin and eosin staining findings

**Figure 1:**
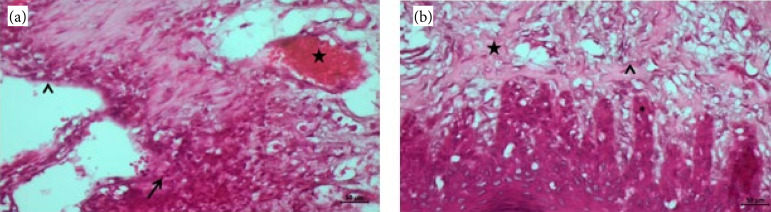
Hematoxylin and eosin staining of the gingival sections. **(a)** Burn group; **(b)** burn + ellagic acid group.

Source: Elaborated by the authors.

In burn group, we observed degeneration (arrowhead) in the epithelium, edema, and inflammatory cell infiltration (arrow) in connective tissue areas, and dilatation and congestion (star) in blood vessels were detected (Fig. 1a). In burn + EA group, improvement in the gingival epithelium (asterix), increase in collagen fiber production (star), uneven distribution of collagenized structures in the dermis (arrowhead) were observed (Fig. 1b).

#### Fibroblast growth factor immunostaining findings

**Figure 2:**
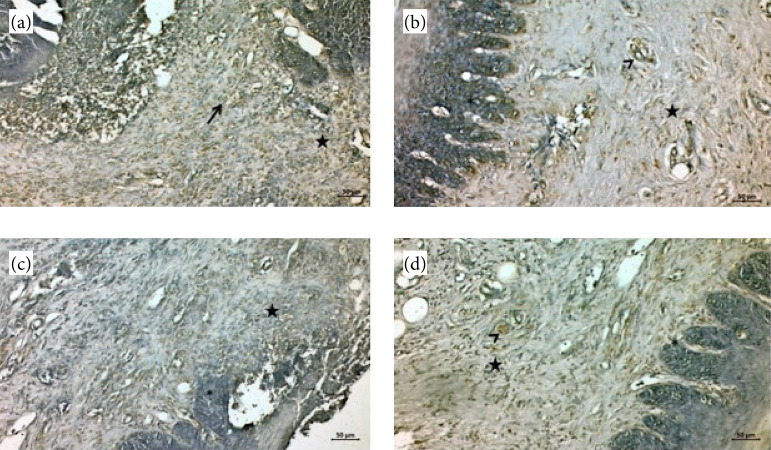
Immunohistochemical staining of the gingival sections. (a and b) Fibroblast growth factor and **(c and d)** epidermal growth factor immunostaining of the gingival sections **(a)** Burn group; **(b)** burn + EA group; **(c)** burn group; **(d)** burn + EA group.

Source: Elaborated by the authors.

In burn group, FGF expression was positive in fibroblast cells (arrow) and other cells of the dermis (star) (Fig. 2a). In burn + EA group, FGF expression was high in the epithelial layer (asterix), dermis (star) and blood vessels (arrowhead) (Fig. 2b).

#### Epidermal growth factor immunostaining findings

In burn group, it was observed that EGF immune activity increased in the gingival epithelium (asterix) and dermis layer (asterisk) (Fig. 2c). In burn + EA group, EGF expression was positive in germinative epithelial cells (asterix), connective tissue (asterisk), and blood vessels (arrowhead) (Fig. 2d). When we compared it with the burn group, it can be said that epithelial structures and collagen fibers began to form.

## Discussion

In this experimental study, the protective effect of EA on gingival tissue was evaluated histopathologically in terms of fibroblast and epidermal growth factors.

EA is an amphiphilic compound with antibacterial and wound-healing properties. It has two lactone rings and four hydroxyl groups to form hydrophilic moiety. This hydrophilic part of EA provides it to involve in the cellular activities. Hydrophilic part also has hydrogen bonding acceptor and donor sites. Together, they make EA biologically active^22^^,^ 23.

There are many applications of EA. A study showed that utilization of water-soluble EA can remove divalent ionic metal ions from aqueous solutions, which shows EA has metal chelants^24^. Another study showed that EA is suitable for polymer applications^25^. Frayne et al. found that EA assemblies attached to CdSe nanoparticles were thermally stable and degraded alizarin red, a model toxic aromatic compound, more efficiently than CdSe nanoparticles. Therefore, the construction of such nanocomposites that can not only bind to environmental pollutants but also efficiently photodegrade them could open new doors to a new class of highly stable, luminescent materials capable of degrading toxic compounds for environmental remediation^26^. In an in-vitro study, Kim et al. investigated a local drug delivery system with chitosan–EA (Ch-EA) film, an implantable polymeric device. It has been stated that the local delivery system they applied consists of chitosan as a polymeric carrier and EA as an anticancer drug. They observed that, with increasing EA concentration, the films became rougher and hydrophilic, and new crystalline peaks of EA were observed. They suggested that, in the range of Ch-EA 0.5 and Ch-EA1, Ch-EA films had a strong antiproliferative effect on melanoma cells in a short time and induced apoptotic cell death^27^.

Basic FGF was first identified in brain and pituitary extracts based on its effects on the proliferation of 3T3 fibroblast cells. Human bFGF was initially thought to be synthesized as a 155 amino acid protein which shares a high degree of sequence homology (98.7%) with bovine bFGF, indicating that it is highly conserved across species^28^. Human bFGF is encoded by one gene mapped to chromosome 4^29^^,^ 30. bFGF has since been identified as an 18 kDa protein with several high molecular weight (HMW) (22–24 kDa) translational variants^31^^,^ 32. Crystallographic analysis of the bFGF protein revealed that the structure is entirely composed of β-sheets. The tertiary structure of bFGF is composed of three 4-stranded β-meander motifs to create a barrel which is closed at the animo- and carboxy-terminus^33^. 18 kDa bFGF is primarily found in the cytosol and increases cell growth and migration, whereas HMW bFGF is primarily localized to the nucleus and increases cell growth, but not migration, and is required for cell growth in low serum conditions^34^^,^ 35.

EGF is a polypeptide that regenerates epidermal cells. Its action is manifested not only at the cellular, but also at the molecular level. It is expressed in slowing down the aging of the skin. The EGF was studied and discovered back in the 1960s by American professor Stanley Cohen. His discovery was highly appreciated, and in 1986 he was awarded the Nobel Prize in Physiology or Medicine as a sign of this. Nowadays, this factor has received the widest application in many areas of medicine and cosmetology^36^.

The gingiva tissue is constantly subjected to mechanical and bacterial aggressions. Unsuccessful gingival healing can lead to scar formation and impaired growth of the palate and dentomaxillary complex^1^. Therefore, to reduce impaired healing following oral surgery, there are ongoing research projects to improve this situation.

Alternative strategies are being investigated in order to promote oral wound healing and tissue regeneration in conjunction with surgical intervention. Different new therapeutics were investigated as a treatment option with the use of synthetic polymers, biological grafts, gel-like ointments, hybrid scaffolds, and drugs, cells, tissue, or growth factors to enhance oral wound healing. These investigations have analyzed the success of oral wound healing therapy using histology for tissue re-epithelialization and microscopy images for wound closure.

Umeki et al.^37^ examined the role of leptin, a naturally occurring hormone in saliva, for the treatment of oral wounds by topically applying it to gingival wounds in Japanese white rabbits. These wounds were 5 mm in diameter and created by the application of filter paper endue in 50% acetic acid to gingival tissue in the mandible. Lim et al.^38^ studied the topical treatment of 1% curcumin in rabbits with a 6-mm gingival wound created using 15 μL of 50% acetic acid. In contrast, Kılıç et al.^39^ performed a 15-mm injury via surgical excision of gingival tissue. They made a five-day study using rabbits to test the effectiveness of local glutathione and chitosan application on adverse healing and reducing oxidation at the wound site.

Another study on aucubin has demonstrated a significant decrease in inflammatory cells at day 5 in aucubin-treated mice compared to control. Masson’s trichrome and picrosirius red were applied, and results showed a significant increase in newly accumulated collagen near the healed area of aucubin-treated mice compared to control at day 5 post-injury^40^. In our study, burn injury significantly decreased GSH, epithelization, FGF and EGF expression. MDA, MPO, inflammation and leukocyte infiltration were significantly increased in burn group. EA treatment improved these scores (Table 1).

Our histopathology results showed degeneration epithelium, edema and inflammatory cell infiltration in connective tissue areas, and dilatation and congestion in blood vessels (Fig. 1a). In burn + EA group, the gingival epithelium improved, collagen fiber production increased and organized dermis were observed (Fig. 1b). FGF expression was positive in fibroblast cells and other cells of the dermis (Fig. 2a). FGF expression was high in the epithelial layer, dermis, and blood vessels (Fig. 2b). It was observed that EGF immune activity increased in the gingival epithelium and dermis layer (Fig. 2c). EGF expression was positive in germinative epithelial cells, connective tissue, and blood vessels (Fig. 2d).

As limitations of the study, the number of animals in the experimental groups in the study was quite small. Experiments with larger numbers of groups and animals would yield safer results. The study was mostly histopathological, but it could have been supported with different techniques and in-vitro studies.

## Conclusion

It was observed better quality of tissue remodeling with the evaluation of regenerative growth factors; EGF and FGF, immunohistochemically. According to our observations, we may suggest that EA have the potential for better healing outcomes in oral wounds. EA seems to have promising therapeutic efficacy to enhance oral wound healing.

## Data Availability

All data sets were generated or analyzed in the current study.
